# The Anti-inflammatory Effects of HMGB1 Blockades in a Mouse Model of Cutaneous Vasculitis

**DOI:** 10.3389/fimmu.2020.02032

**Published:** 2020-09-29

**Authors:** Jin Wang, Lixin Fu, Hao Yang, Kai Cao, Qiaomei Sun, Tao Chen

**Affiliations:** Department of Dermatovenereology, Chengdu Second People's Hospital, Chengdu, China

**Keywords:** high mobility group box-1, glycyrrhizin, reverse passive arthus reaction, vasculitis, inflammation

## Abstract

In our previous study, we have found increased serum levels of HMGB1 in patients with Henoch– Schonlein purpura (HSP), allergic vasculitis (AV), and urticarial vasculitis (UV) and altered HMGB1 distribution in lesional skin in patients with HSP. HMGB1 plays a pro-inflammatory role in the pathogenesis of HSP. To further investigate the role of HMGB1 in the pathogenic mechanism of vasculitis, we investigated the anti-inflammatory effects of HMGB1 blockades (including anti-HMGB1 mAb and glycyrrhizin) in a mouse model of a cutaneous reverse passive Arthus (RPA) reaction. A total of 36 balb/c mice were randomly divided into four groups: the control group, IC model group, HMGB1 monoclonal antibody (anti-HMGB1-mAb) group and the glycyrrhizin group, with nine mice in each group. A cutaneous RPA reaction mouse model was established by injections of the OVA antibody and the OVA antigen. Mice of the anti-HMGB1-mAb group and glycyrrhizin group were pre-treated with anti-HMGB1 mAb or glycyrrhizin, respectively, before the RPA reaction. Our results indicated that HMGB1 blockades (anti-HMGB1 mAb and glycyrrhizin) obviously extenuated the severity of vasculitis skin damage and improved the histological evolvement of inflammatory cells infiltration, vascular fibroid necrosis, and vasodilation in a cutaneous RPA reaction mouse model. In addition, HMGB1 blockades reduced the infiltration of neutrophils, DCs, and T cells and decreased the mRNA expression of IL-6 and CCL5 in skin lesions in the cutaneous RPA reaction mouse model. We suggest that HMGB1 blockades may represent a new direction for the treatment of cutaneous vasculitis.

## Introduction

Vasculitis, that can occur in all sizes and types of blood vessels in almost all organs, is a procedure of clinical pathology, characterized by an infiltration of inflammatory cytokines around the blood vessel wall and blood vessels. Cutaneous vasculitis (CV) may be the most representative symptom of the varying degrees of vasculitis or may be the part most associated with other primary systemic diseases. It has been found in histology that CV is intimately related to immunopathological mechanisms and may be caused by immune cells (such as neutrophils, lymphocytes, or eosinophils) that mediate inflammation (1–3). It is well-established that the production and release of proinflammatory cytokines such as TNF- α and IL-6 play crucial roles in IC-induced inflammation (4–6).

High mobility group box 1 (HMGB1), which is described as a highly conserved non-histone DNA-binding protein, was discovered to be a crucial cytokine that mediates the response to infection, injury, and inflammation (7, 8). Antigen presenting cells (APC) can activate immune responses against pathogens. With the absence of pathogens, endogenous molecules (e.g., HMGB1 is passively released from necrotic cells or secreted by stressed cells to respond to cellular injury) activate APCs, resulting in autoimmune diseases and transplant rejections (9, 10). It has been proven in our previous study that HMGB1 is involved in the pathogenesis of inflammatory and autoimmune disorders, such as, Henoch– Schonlein purpura (HSP), allergic vasculitis (AV), and urticarial vasculitis (UV). It also altered HMGB1 distribution in lesional skin in patients with HSP (11–13). However, the function and mechanism of HMGB1 in vasculitis has not been clearly stated.

Glycyrrhizin is an active ingredient of licorice, and can be extracted or chemically synthesized (14). Glycyrrhizin has been confirmed to have both anti-inflammatory and anti-viral influences by combining directly to HMGB1, and inhibits its chemoattractant and mitogenic activities (15). Our previous study found that glycyrrhizin suppresses TNF- α induced chemokine production in HMEC-1 cells (16). We also found that the serum HMGB1 level of 16 Henoch– Schonlein purpura patients was significantly lower after treatment with glycyrrhizin (11).

Cutaneous reverse passive Arthus (RPA) reaction is a classical animal model of CV, in which immune-complex-induced endothelial inflammatory responses play essential roles (11). In our research, the anti-inflammatory effects of HMGB1 blockades stems from an understanding of the biological basis of the HMGB1 inflammation, and we further investigate the role of HMGB1 in the pathogenic mechanism of vasculitis in a mouse model of cutaneous reverse passive Arthus (RPA) reaction.

## Materials and Methods

### Mice

Thirty-six balb/c mice aged from 6 to 8 weeks old were chosen in this study from the Sichuan University Animal Center (Sichuan, China) with free access to drinking water and food. All animal procedures were approved by the Institutional Animal Care and Use Committee of Chengdu Second People's Hospital and carried out in accordance with guidelines for the Care and Use of Laboratory Animals of National Institute of Health.

### Animal Model

A total of 36 balb/c mice were randomly divided into four groups: the control group, IC model group, HMGB1 monoclonal antibody (anti-HMGB1-mAb) group and glycyrrhizin group, with nine mice in each group. At the beginning of the experiment, the mice of the anti-HMGB1-mAb group were injected i.p. with anti-HMGB1 mAb (Sino Biological, Beijing, China) 2 mg/kg once every other day three times. For the glycyrrhizin group, mice were injected i.p. with glycyrrhizin (20 mg/kg; Nippon Kayaku, Tokyo, Japan) once every day for 6 days. The mice of the control group and IC model group, an equal volume of phosphate-buffered saline (PBS) was injected i.p. once every day for 6 days.

A mouse model was established on the last day of treatment mentioned above in each group. Mice were subjected to intradermal injection of the OVA antibody. Tail vein injections were given with the OVA antigen, some mice were injected by tail vein with the OVA antigen and 1% Evan's blue solution. Mice in the control group were given an intradermal injection with PBS solution on the back. The lesional skin on the back of the mice and the extent of exudation of 1% Evan's blue solution were observed. After that, mice were euthanized. Tissue samples in different groups were obtained.

### Histological Examination and Immunohistochemical Staining

According to standard techniques, hematoxylin–eosin (HE) staining was performed on tissue sections of histological observation and immunohistochemistry with CD3 (Abcam, Cambridge, UK), CD11c (Beijing Biosynthesis, Beijing, China) and myeloperoxidase (MPO; Beijing Biosynthesis, Beijing, China). Morphological changes of skin lesions were observed by light microscopy (BX60; Olympus, Tokyo, Japan).

### Real-Time Quantitative Polymerase Chain Reaction

Real-time quantitative polymerase chain reaction (qPCR) tests were taken to detect the mRNA expression of IL-6 and CCL5 in the skin of every group. Total RNA in every groups was extracted from the Trizol reagent (Invitrogen Corp, Carlsbad, CA, USA) in conformity with the manufacturer's instructions. In the established model system, qPCR primers and the SYBR Green Master Mix were used to carry out the experiments. The following primers of IL-6 (MQP036632; GeneCopoeia, Rockville, MD, USA) and CCL5 (MQP030981; GeneCopoeia, Rockville, MD, USA) were used. In our established model, the reference gene and glyceraldehyde 3-phosphate dehydrogenase were analyzed by way of 2 ^ΔΔ*CT*^ and the expression levels and fold changes of these cytokine controls were analyzed. GAPDH was used as the internal reference gene. The reaction conditions in this experiment were: pre-denaturation at 95°C for 3 min, denaturation at 95°C for 15 s, annealing at 58°C for 30 s, and extension at 72°C for 30 s for a total of 40 cycles.

### Statistical Analysis

Mean ± SD were used for all results; one-way analysis of variance was used for statistical differences between groups. Data were analyzed with the Graphpad Prism software (GraphPad Sotware, La Jolla, CA, USA). Statistical significance was accepted at the level of *P* < 0.05. All experiments were carried out at least three times.

## Results

### HMGB1 mAb and Glycyrrhizin Obviously Extenuated the Severity of Vasculitis Skin Damage

**Figure 1:**
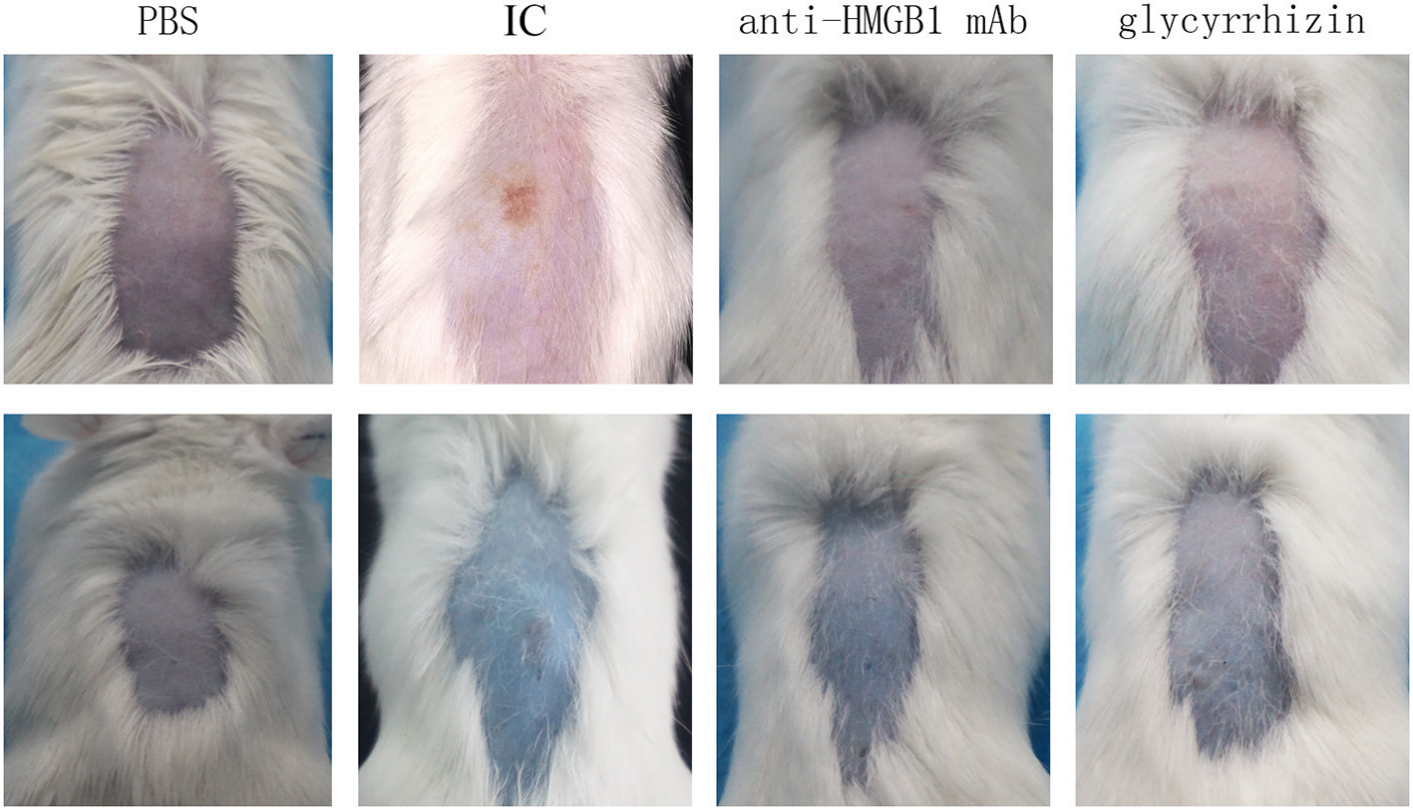
Mice were injected i.p. with PBS, anti-HMGB1 monoclonal antibody, or glycyrrhizin, respectively. Mice were subjected to intradermal injection of the OVA antibody. Tail vein injections were given with the OVA antigen and 1% Evan's blue solution. Mice in the control group were given an injection with the PBS solution on the back. The macroscopic presentation of mice back skin was shown. IC showed obvious vasculitic lesions and Evan's blue exudation on the back contrasting with the other groups. IC, model control group; anti-HMGB1 mAb, anti-HMGB1 monoclonal antibody. *n* = 9 per group.

We observed skin lesions in all groups on the back of the mice and the extent of exudation of 1% Evan's blue solution. As presented in Figure 1, compared with the PBS control group, the model group showed obvious vasculitic lesions and Evan's blue exudation on the back. While treatment with the HMGB1 monoclonal antibody and glycyrrhizin, the skin on the back of the mice was significantly reduced in the local inflammatory response compared with the model group.

### Significant Improvement in Inflammatory Cells Infiltration, Vascular Fibroid Necrosis, and Vasodilation Treated With the HMGB1 Blockade

**Figure 2:**
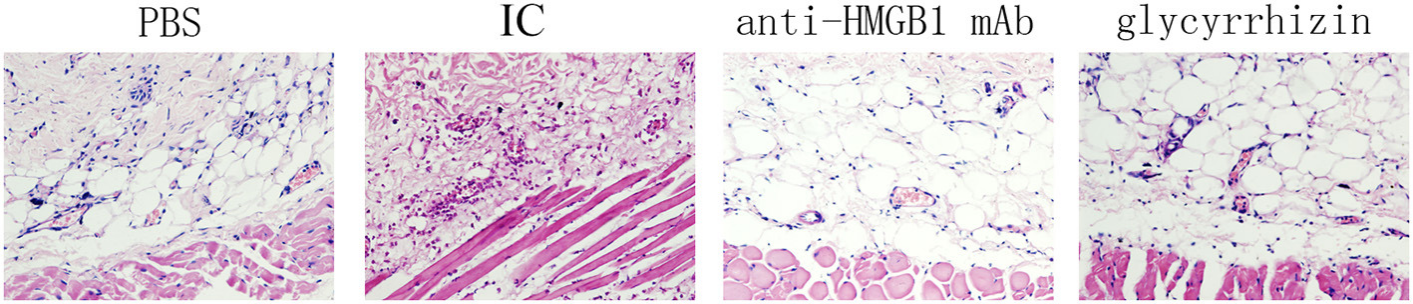
Shown as a histological examination, Phenotypical presentation of mouse back skin which was injected with PBS, anti-HMGB1 monoclonal antibody, or glycyrrhizin, respectively, were applied and hematoxylin–eosin (HE) staining of tissue sections treated as described. There was more inflammatory cell infiltration, vascular fibroid necrosis, and vasodilation in the model control group, compared with the other groups. *n* = 6 per group.

Morphological changes of skin lesions were observed by light microscopy. During the histological examination, in contrast with the PBS group, we found that there was more inflammatory cell infiltration, vascular fibroid necrosis, and vasodilation in the model group, nevertheless, there was a significant improvement in the tissue treated with the HMGB1 monoclonal antibody and glycyrrhizin as seen in Figure 2.

### HMGB1 Blockade Reduces Inflammatory Cell Infiltration in Vasculitis Mice Model

**Figure 3:**
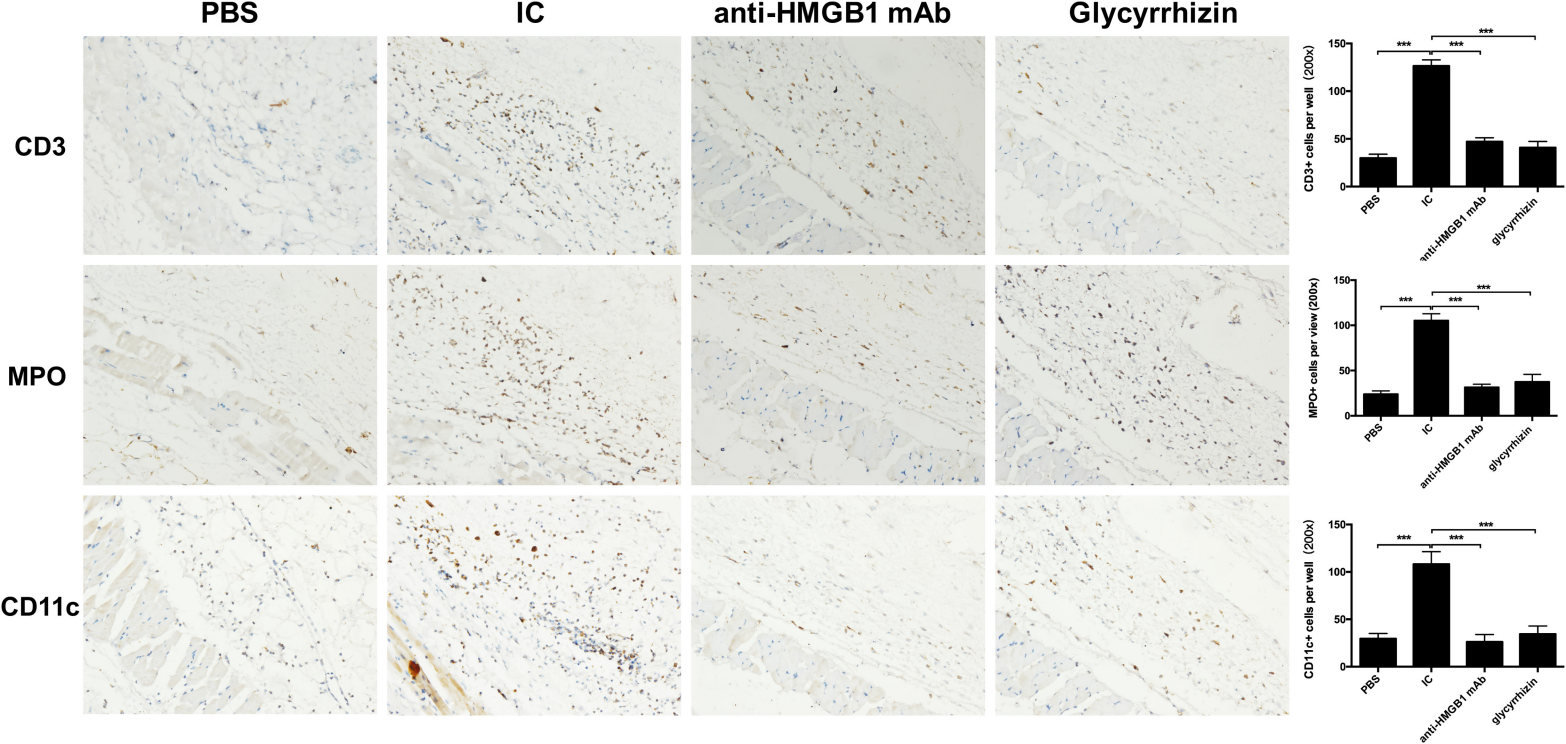
Results of immunohistochemical staining of four groups in CD3, MPO, and CD11c. Image shows high power (200x). More inflammatory cell infiltration, vascular fibroid necrosis, and vasodilation in the IC when compared with the other groups. *n* = 6 per group. ^*^P < 0.05, ^**^P < 0.01, and ^***^P < 0.0001.

Immunohistochemistry was adopted to analysis the infiltration of the inflammatory cells. In flagrant contrast with PBS group, there was a marked reduction of lymphocytes, neutrophils, and dendritic cells infiltrating the model group. However, compared with the model group, the above inflammatory cells were significantly reduced after treatment with the HMGB1 antibody and glycyrrhizin as shown in Figure 3.

### The mRNA Expression of IL-6 and CCL5 Were Reduced Evidently in the HMGB1 Monoclonal Antibody Group and Glycyrrhizin Group

**Figure 4:**
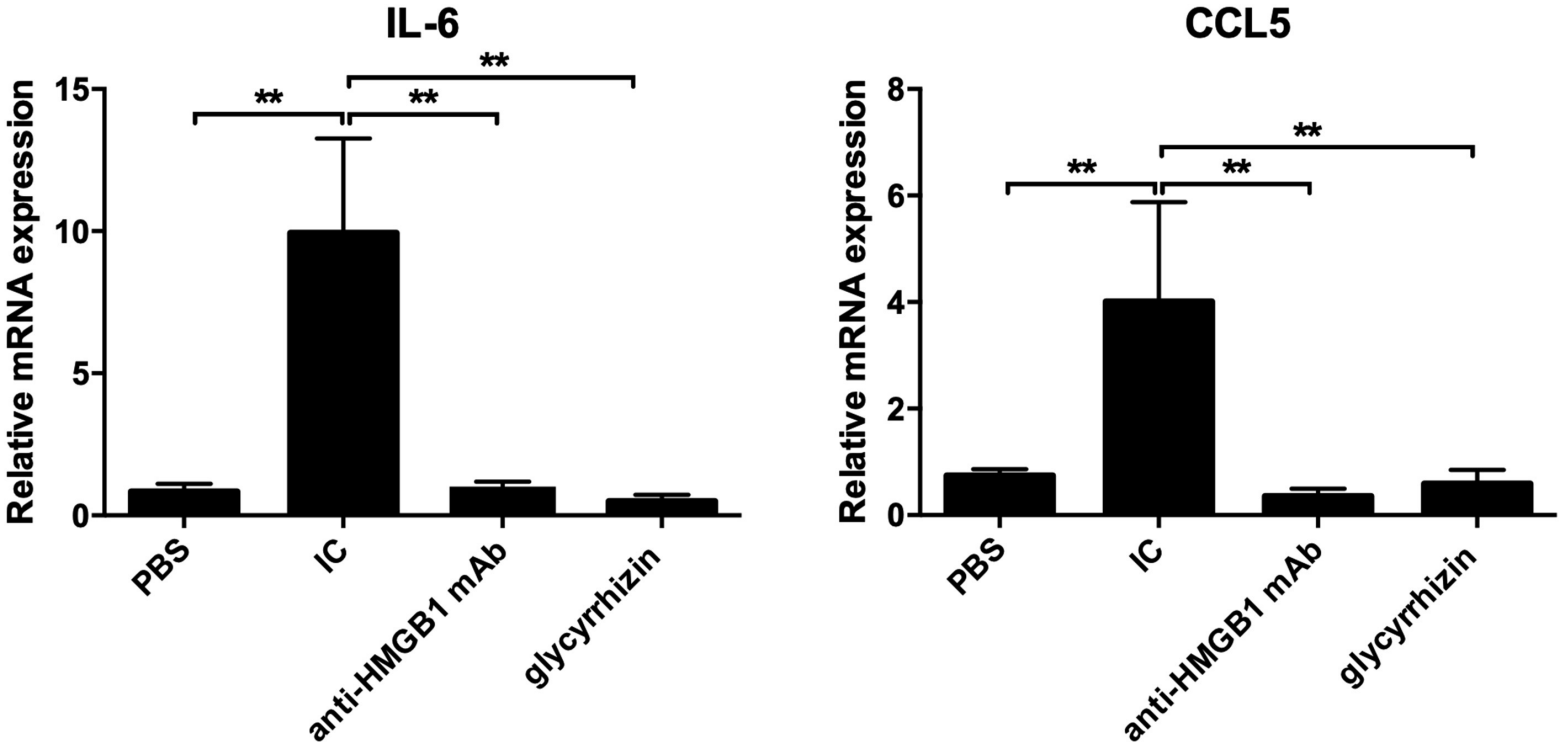
PCR was taken to detect the mRNA expression of IL-6 and CCL5. Total RNA were extracted from the Trizol reagent. In contrast with PBS, the mRNA level of IL-6 and CCL5 was markedly higher in IC, yet there were reduced in other groups. *n* = 6 per group. ^*^P < 0.05 and ^**^P < 0.01.

As displayed in Figure 4, compared with the PBS group, the results of qPCR demonstrated that the mRNA expression of IL-6 and CCL5 had reduced evidently in the scathing tissue of the HMGB1 monoclonal antibody group and glycyrrhizin group (*P* < 0.05), yet there was a marked increase in the control group.

## Discussion

The high mobility group box 1 protein (HMGB1) is universal in all the cells of higher eukaryotes, is secreted by inflammatory cells (such as: activated monocytes, macrophages, mature dendritic cells, and natural killer cells), and may act as a potent mediator of inflammation (17–20). Our previous work found that the expression of HMGB1 was elevated in a variety of vasculitis diseases, and it was decreased in patients with HSP after treatment (13). HMGB1 may play an important role in the pathogenic mechanism of vasculitis. In this study, a HMGB1 blockade was used to treat vasculitis in a mouse model of cutaneous vasculitis. Then we detected that local skin lesions and tissue inflammatory cell infiltration were both significantly reduced. It meant that a HMGB1 blockade can effectively improve the clinical and pathological manifestations of vasculitis in mice.

It is well-established that the production and release of proinflammatory cytokines such as TNF- α and IL-6 play crucial roles in IC-induced inflammation (4–6). In this study, we found that the expression of related inflammatory cytokines IL-6 and CCL5 were markedly increased in the skin samples of mice from the IC group compared with the control group.

The HMGB1 protein is not only a nuclear factor but also a secreted protein (8). It can stimulate activated monocytes, neutrophils, and the production of IL-7, IL-8, TNFα, MMP, and other pro-inflammatory cytokines, and can also promote the development of various chemokines. HMGB1 can also promote the local production of the tumor-necrosis factor (TNF), interleukin-6 (IL-6), and interferon-γ (7, 21). HMGB1 can induce phosphorylation of the inhibitor of κB-a (IκBa) and the nuclear translocation of nuclear factor-κB (NF-κB) p65 in HMEC-1 cells. The signaling pathway leads to the activation of NF-κB, which leads to an increased expression of CCL5, which in turn triggers a subsequent inflammatory response, resulting in more inflammatory cells infiltrating the damaged tissue, further aggravating pathological damage (18, 22). Maeda et al. (23) found that anti-HMGB1 antibodies inhibited the production of TNF-α and IL-6 by blocking extracellular HMGB1. In order to find out whether it reduced the inflammatory response by inhibiting HMGB1, we used anti-HMGB1 mAb and the HMGB1 blocker compound glycyrrhizin to treat the mice model. Then, we found the expression of related inflammatory cytokines IL-6 and CCL5 were significantly decreased after treatment, it may further reduce the damage of vasculitis. That means that a HMGB1 blockade can effectively inhibit the infiltration of inflammatory cells and the expression of inflammatory cytokines in the skin lesions of vasculitis mice (18, 19).

Previous studies suggest that glycyrrhizin has anti-inflammatory, antiviral, antimicrobial, antioxidative, anticancer activities, and immunomodulatory effects (24, 25). In Japan, a glycyrrhizin preparation called Stronger Neo-Minophagen C (SNMC) has been used as an anti-allergic and anti-hepatitis agent in clinical treatment for 60 years (26, 27). It has been reported that glycyrrhizin can effectively inhibit the cytoplasmic transduction of HMGB1. Our previous studies also found that glycyrrhizin can target the inhibition of cytoplasmic transduction in HMGB1 and thus inhibit the expression of inflammatory cytokines, and it has been proven that glycyrrhizin is a direct inhibitor of HMGB1 (13). This study used glycyrrhizin to effectively treat vasculitis mice and reduce inflammation. It was once again demonstrated that glycyrrhizin acts as an inhibitor of HMGB1 and also has an inhibitory effect on vasculitis.

In general, we have experimentally demonstrated that HMGB1 is closely related to the pathogenesis of vasculitis, and that the HMGB1 blockade can significantly improve the inflammatory response of vasculitis, resulting in a more effective, safe, and potentially new treatment for vasculitis.

## Data Availability Statement

All datasets generated for this study are included in the article/supplementary material.

## Ethics Statement

The animal study was reviewed and approved by Institutional Animal Care and Use Committee of Chengdu Second People's Hospital.

## Author Contributions

LF and TC: concept, design, and definition of intellectual content. JW and LF: literature search. LF, JW, and KC: experiment studies. QS and HY: data acquisition and data analysis. JW and LF: manuscript preparation. JW, LF, and TC: manuscript editing. TC: manuscript review. All authors contributed to the article and approved the submitted version.

## Conflict of Interest

The authors declare that the research was conducted in the absence of any commercial or financial relationships that could be construed as a potential conflict of interest.
